# Enhancing Flavor in Dried Mackerel Floss (*Scomberomorus niphonius*) via Protease: Formation Mechanism of Characteristic Flavor Revealed by Integrated Multi-Omics Analysis

**DOI:** 10.3390/foods14111864

**Published:** 2025-05-24

**Authors:** Diqian Yang, Xiaohui Li, Haowei Wu, Runyu Tang, Qiuying He, Huanhuan Dai, Weiqiang Qiu

**Affiliations:** 1 College of Food Science and Technology, Shanghai Ocean University, Shanghai 201306, China; 18781687087@163.com (D.Y.); wuhaowei906@outlook.com (H.W.); 18117318531@163.com (R.T.); heqiuying1122@163.com (Q.H.); 2 National Experimental Teaching Demonstration Center for Food Science and Engineering, Shanghai Ocean University, Shanghai 201306, China; 3 Shanghai Aquatic Product Processing and Storage Engineering Research Center, Shanghai Ocean University, Shanghai 201306, China; 4 Department of Life Science, Hefei Normal University, Hefei 230061, China; daihuanhuan1987@126.com

**Keywords:** mackerel, fish floss, protease, flavor, multi-omics, HS-GC-IMS, volatile organic compounds, free amino acids

## Abstract

Current marine mackerel (*Scomberomorus niphonius*) products predominantly involve low-value-added processing, while high-value-added products like fish floss remain underdeveloped. This study utilized mackerel dorsal muscle treated with flavor protease (FP), papain (PP), and neutral protease (NP) (10 U/g, 30 min), followed by steaming and stir-frying. Combined with sensory evaluation, HS-GC-IMS, and automatic amino acid analysis, the characteristic flavor was evaluated by multi-omics. The results showed that FP and NP significantly enhanced odor by reducing fishy compounds (e.g., hexanal) and increasing pyrazines/furans. PP enhanced taste by elevating umami and sweet amino acids (26.68% and 25.98%, respectively). Correlation analysis revealed the following potential pathways: Val and Leu served as precursors for furan, suppressing 2-methyl-3-(methylthio)furan formation, while Asp, Tyr, Phe, Gly, Cys, and Ile promoted 2,5-dimethylpyrazine and 2-methyl-3-(methylthio)furan generation while inhibiting furan. This study demonstrates that minimal protease addition effectively optimizes dried mackerel floss flavor, providing a novel approach for high-quality marine product development.

## 1. Introduction

Mackerel (*Scomberomorus niphonius*) is widely distributed in the temperate waters of the northwest Pacific Ocean, serving as one of the most important fishery resources for multiple countries, including China [1]. Current research on mackerel processing technologies has been primarily focused on surimi products [2], dry-salted marinated products (DSM) [3,4], and other primary processed goods, with relatively low processing efficiency and added value. The development of refined products with high value-added, such as dried mackerel floss (DMF), could improve resource utilization and product commercial potential [5]. Existing fish floss production mainly focuses on the use of freshwater fish and miscellaneous fish caught in the ocean as raw materials [6]. Marine species possess unique nutritional advantages over freshwater counterparts, containing abundant essential macro/micronutrients and highly bioavailable long-chain omega-3 polyunsaturated fatty acids [7]. Nevertheless, currently, the market for highly processed foods and marine foods lacks a diverse range of products. Coupled with the growing consumer preference for fish floss, there is an urgent need for research on related products to fill this market gap. As a type of marine fish product, DMF shows promising prospects for market development.

Biological enzymatic hydrolysis technology has the advantages of being environmentally friendly, highly efficient, having mild reaction conditions, and offering high safety and controllability [8]. It is frequently employed in the processing of aquatic products and soy products to enhance the flavor of foods and improve the utilization rate of raw materials. Currently, most research on fish focuses on the extraction and isolation of waste materials. For instance, umami peptides have been successfully separated from seafood such as oysters [9], scallops [10], and large yellow croakers [11]. Moreover, flavor proteases and papain have been employed in the enzymatic hydrolysis of *Trachinotus ovatus* muscle tissues to obtain protein hydrolysates with low bitterness, high freshness, and saltiness [12,13].

In addition, although enzymatic digestion has demonstrated potential for aroma improvement in a variety of foods, such as cheese and fish [14], few studies have been reported on enzymatic digestion of the raw material itself, which then undergoes a series of reactions during the cooking process to achieve enhancement of the flavor of the food itself.

Enzymatic hydrolysis can facilitate the breakdown of proteins and lipids, thereby generating volatile aroma precursors for lipid oxidation and Maillard reaction (MR) [15]. For example, nucleophilic amine groups in amino acid residues can interact with the carbonyl groups of sugars, which can produce volatile aromatic substances and generate melanoidins, thus further improving the color and flavor of foods [16,17], Additionally, the free amino acids (FAAs) released during hydrolysis act as flavor-presenting substances and can also provide a certain taste. Previous studies have elucidated the formation pathways of pyrazines through the establishment of methionine/glucose (Met/Glc) and Amadori rearrangement product models derived from Met/Glc [18]. Additionally, enzymatic hydrolysates of animal fats can produce a characteristic meaty aroma through a reaction system with sugars and amino acids [15,19,20]. Moreover, the addition of different sugars significantly affects the odor profile of fish floss.

As a kind of refined food, the flavor development of fish floss during frying is primarily influenced by multiple factors, including MR and lipid oxidation [5], and these studies have demonstrated the feasibility of moderately enzymatic hydrolysis of fish meat for the preparation of fish floss and provided some theoretical basis.

In recent years, multi-omics analysis of food products has increasingly employed advanced instrumental techniques, such as an electronic nose (E-nose), GC-MS, GC-IMS, and automatic amino acid analyzers, to characterize dynamic variations in volatile organic compounds (VOCs) and FAAs [21,22,23,24]. These instrumental detection technologies offer distinct advantages, including cost-effective operation, minimal sample pretreatment requirements, rapid throughput, and high sensitivity. They also have great potential in predicting flavor formation mechanisms, which are widely used in the global food industry [21].

As mentioned above, this study hypothesizes that the use of different proteases for hydrolysis may significantly improve the odor or taste characteristics of DMF through lipid oxidation and MR. To investigate this hypothesis, the present study was designed with two primary objectives: (1) to significantly enhance the flavor profile of DMF treated with different proteases, and (2) to reveal the formation mechanisms of the characteristic flavor substances.

To achieve these objectives, dorsal muscle tissue of mackerel was utilized as the substrate. The tissue was subjected to moderate enzymatic hydrolysis using flavor protease (FP), papain (PP), and neutral protease (NP). The hydrolysates were then processed into fish floss. To determine the flavor-enhancing effect of this processing method on DMF, the characteristic flavors were systematically characterized using an E-nose, HS-GC-IMS, and an automatic amino acid analyzer, complemented by multi-omics analysis. Furthermore, correlation analysis between VOCs and FAAs was performed to elucidate the underlying mechanism governing flavor formation. This investigation established a theoretical framework for the preparation and flavor evaluation of DMF, while proposing an innovative approach to enhancing its flavor through enzymatic treatments.

## 2. Materials and Methods

### 2.1. Sample Preparation

**Figure 1:**
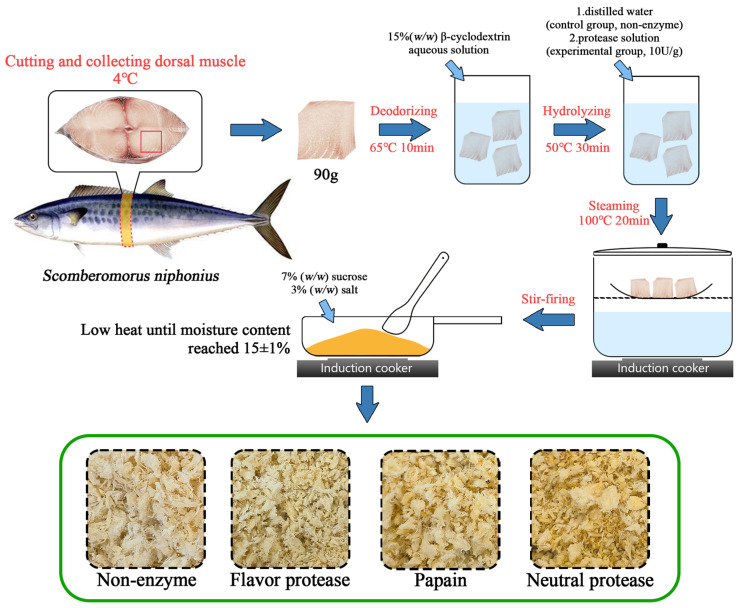
Flowchart of the preparation of dried mackerel floss (DMF).

Mackerel *(Scomberomorus niphonius)* was purchased from a fresh food supermarket in Shanghai, China, and stored at −18 °C. The frozen mackerel was thawed, after which the fish was repeatedly washed with water. The head, tail, internal organs, and dark muscle were removed, and 90 g of dorsal muscle was collected. The fish muscle was immersed in an aqueous solution of 15% (*w*/*w*) β-cyclodextrin at 65 °C for 10 min to deodorize the fish. Following this, the fish muscle was either immersed in protease solutions (10 U/g) at 50 °C for 30 min or in distilled water (non-enzyme) for 30 min. The material–liquid ratio (fish mass:water) was 1:2, and the different proteases were flavor protease (FP, 15,000 U/g), papain (PP, 10,000 U/g), and neutral protease (NP, 50,000 U/g). Afterward, the fish muscle was steamed at 100 °C for 20 min and torn along the muscle fibers; 7% (*w*/*w*) sucrose (AR) and 3% (*w*/*w*) salt were added; and it was stir-fried over moderate heat to loosen the fish until the moisture content reached 15 ± 1%. A flowchart of making DMF is shown in Figure 1.

### 2.2. Sensory Evaluation

The sensory evaluation group consisted of 10 members (6 males and 4 females, between 20 and 30 years old) who had undergone professional training. The evaluation indexes included texture, color, odor, and taste, according to GB/T23968-2022 (“General quality for meat floss”) [25]. All members conducted their assessments in identical environmental conditions and completed standardized scoring questionnaires immediately after each assessment. Based on the sensory evaluation form used by Huo et al. [26], with appropriate modifications, the weights assigned to each standard and the detailed scoring rules are presented in Table 1. The formula for calculating the total score is as follows:

Total score = Texture × 0.10 + Color × 0.20 + Odor × 0.35 + Taste × 0.35(1)

### 2.3. Electronic Nose Measurement

E-nose analysis was conducted using an α-Fox4000, electronic nose (Alpha MOS, Toulouse, France) following a modified protocol based on the method established by Wang et al. [27]. Prior to analysis, the instrument was calibrated and subjected to self-diagnostic tests to ensure operational reliability. Specifically, 1.0 g of each sample was precisely weighed into a 10 mL headspace vial. The samples were incubated at 85 °C for 15 min with continuous stirring at 500 rpm. Automatic sampling was adopted with an injection volume of 2400 μL. High-purity synthetic dry air was used as the carrier gas with a flow rate of 150 mL/min. All measurements were conducted in triplicate to ensure reproducibility. Detailed sensor parameters [28] are presented in Table 2.

### 2.4. Volatile Compound Analysis

The analysis of volatile organic compounds (VOCs) was performed using HS-GC-IMS (FlavourSpec^®^ instrument, G.A.S., Dortmund, Germany) following a modified protocol based on the method established by Li et al. [29]. A 1.0 g sample was weighed into a 20 mL headspace vial. The incubation parameters were set to 85 °C for 15 min. Automated sampling was performed with an injection volume of 500 μL and a needle temperature of 85 °C. Nitrogen was used as the carrier gas, and the separation was achieved using a strong polarity MXT-WAX column (30 m × 0.53 mm, 1 μm). The initial flow rate was 2 mL/min for 2 min, followed by a gradient increase to 10 mL/min over 8 min, and then to 100 mL/min over 10 min, which was held for 10 min, resulting in a total run time of 30 min. All measurements were conducted in triplicate to ensure reproducibility.

### 2.5. Relative Odor Activity Value

The odor activity value (OAV) serves as an indicator of a specific odorant’s contribution; however, due to the complex and diversity of VOCs encountered during measurement, precise absolute quantification presents considerable challenges. Consequently, the relative odor activity value (ROAV) was employed to evaluate odorant contributions.

In this method, the substance with the highest odor contribution was assigned as ROAVmax = 100, while the contributions of other VOCs (ROAVi) were calculated using the following equation [27]:

ROAVi = (Ci × Tmax)/(Ti × Cmax) × 100(2)

where Ci and Cmax represent the relative contents of other VOCs and the dominant odorant contributing most significantly to the overall odor profile, respectively, while Ti and Tmax denote the olfactory thresholds of other VOCs and substances that contribute most to the overall odor.

### 2.6. Free Amino Acids Analysis

Quantitative analysis of free amino acids (FAAs) was performed using an LA8080 automated amino acid analyzer (Hitachi, Japan) following a modified protocol based on the method established by Jin et al. [24]. Briefly, 15 mL of 15% (*w*/*w*) trichloroacetic acid (AR) was added to a 1.0 g sample. The samples were then homogenized (T25 digital ULTRA-TURRAX, IKA, Staufen, Germany) at 6000 rpm for 1 min and then allowed to stand for 2 h. After that, they were centrifuged (MGL-16MT, Merrick, Shanghai, China) for 10,000× *g* at 4 °C for 15 min. A total of 5 mL of the supernatant was taken, and the pH was adjusted to 2.0 with 1 mol/L NaOH (AR) solution. The mixture was then diluted to a final volume of 50 mL with deionized water and thoroughly mixed. The solution was filtered through a 0.22 μm aqueous-phase membrane filter before being transferred to a 2 mL injection vial. Automated sampling was performed with an injection volume of 20 μL. It should be noted that all sample preparation procedures were carried out under controlled temperature conditions (0–4 °C). All measurements were conducted in triplicate to ensure reproducibility.

### 2.7. Taste Activity Value

The taste activity value (TAV), calculated as the ratio of compound concentration to its taste threshold, can reflect the contribution of a flavor substance, and TAV > 1 indicates that the substance is an important flavor substance. The thresholds and calculation formula were adopted from Chen et al. [28]:

TAV = C/T(3)

where C represents the absolute content of the taste substance, and T represents the taste threshold of the substance.

### 2.8. Statistical Analysis

SPSS27 (SPSS Inc., Chicago, IL, USA) was first used for normality tests (Shapiro–Wilk and Kolmogorov–Smirnov), with *p* > 0.05 indicating normal distribution. Only data meeting this normality assumption were subjected to further statistical analyses, including an ANOVA test, Duncan’s multiple comparison, and Pearson’s correlation analysis, with a significance level set at *p* < 0.05 and a highly significant level at *p* < 0.01. Origin 2024 (Northampton, MA, USA) was used to draw column charts, stacked charts, cluster heatmaps, etc. VOCal 0.4.03 was used for the analysis of GC-IMS spectra. The NIST2020 database was used to perform qualitative analysis. The topographic map and subtraction map were viewed using the reporter plugin, and the fingerprint spectra were viewed using the galerie plugin. Principal components analysis (PCA), partial least squares discrimination analysis (PLS-DA), and the variable importance in projection (VIP) were performed on the website Metaboanalyst (https://www.metaboanalyst.ca/ (accessed on 16 February 2025)) after normalization with the non-enzyme group as the standard. Graphic integration and beautification were carried out using Adobe Photoshop 2024 (Adobe Systems, San Jose, CA, USA).

## 3. Results and Discussion

### 3.1. Sensory Evaluation Analysis

The sensory scores of DMF are presented Table 3, and the texture and color characteristics are also presented in the green box of Figure 1, which shows that there was no significant difference in texture of the samples among all groups. In terms of color, NP performed better, with a score of 84.30, showing a significant difference (*p* < 0.05). This might be attributed to the specificity of NP, as the fish muscle generated more melanoidin precursors for melanoidin formation after enzymatic hydrolysis. In terms of odor, FP, PP, and NP were all significantly better than NON (*p* < 0.05). In terms of taste, PP was significantly better than NON, FP, and NP (*p* < 0.05). This is because, after protein enzymatic hydrolysis, aroma precursors and flavor-contributing substances (such as FAAs [24] and umami peptides [11]) were provided for lipid oxidation and the MR. In terms of the total score, FP and PP were also significantly better than NON (*p* < 0.05). These findings suggest that, regardless of which protease was used, the samples showed certain advantages in odor and taste scores, effectively enhancing the flavor of DMF.

Although sensory evaluation is simple to operate and can capture complex sensory experiences, it is highly subjective, and the evaluators are susceptible to sensory fatigue, which may lead to significant variability in the results.

### 3.2. E-Nose Analysis

**Figure 2:**
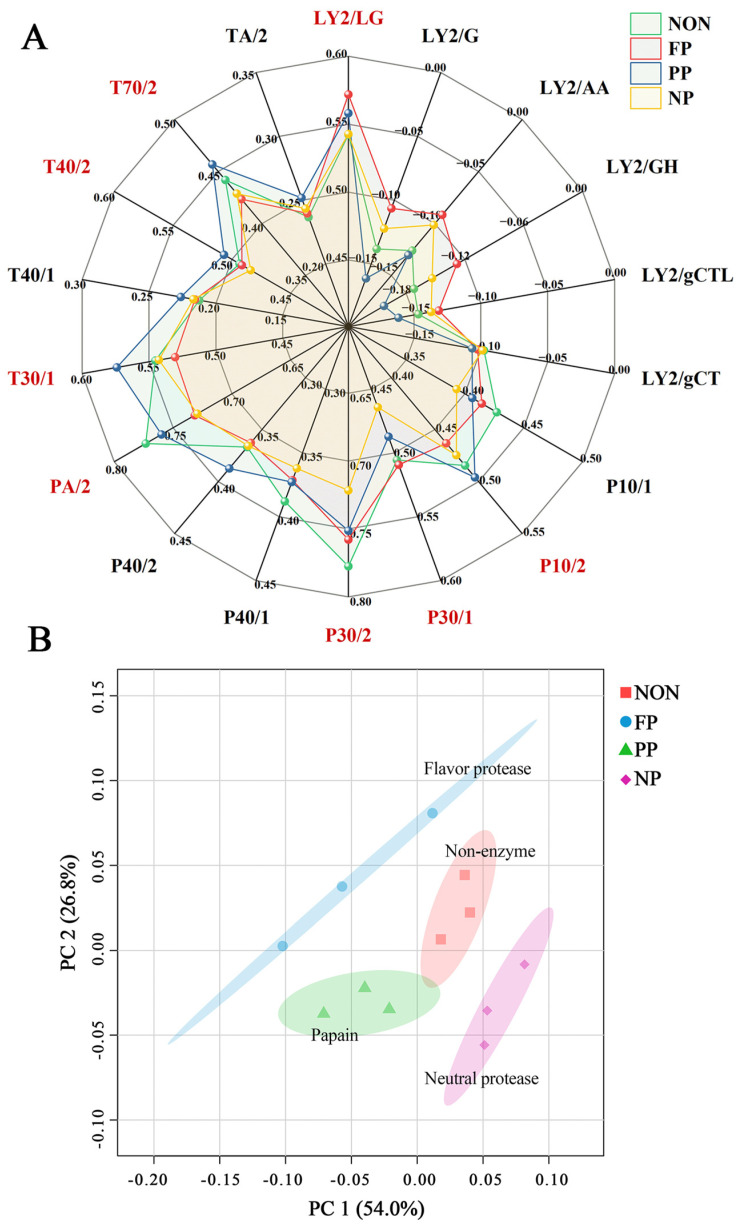
(**A**) Radar plot of E-nose sensor response values in DMF treated with different proteases (sensors highlighted in red indicate response values > 0.45 in at least one treatment group); (**B**) PCA score plot of response values.

An E-nose is an instrument that emulates the human olfactory system through a gas sensor array and pattern recognition algorithms to characterize the VOCs profile of samples. By utilizing sensor-derived data, this approach eliminates potential human bias, thereby ensuring high objectivity. Notably, higher response values indicate a greater content of the corresponding VOCs, rendering them more olfactorily detectable. The E-nose sensors demonstrate selective responsiveness to specific VOCs, with response values ≥ 0.45 being considered valid for reliable detection [27]. Therefore, analysis was restricted to sensors with valid response values. The specific VOCs corresponding to each sensor are listed in Table 2. A radar plot constructed using the response values of each sensor to the samples is shown in Figure 2A (Table S1), where sensors meeting the requirement for valid response values (response value > 0.45) are highlighted in red. Sensors in the P series, such as P10/1 (chloride, fluoride), P30/1 (carbon oxide, ethanol, hydrocarbon, ammonia), P30/2 (aldehyde, ethanol, hydrocarbon, hydrogen sulfide), and PA/2 (ethanol, ammonia, amines), exhibit relatively high response values. This indicates that one or more components among aldehydes, alcohols, and hydrocarbons make significant contributions to the odor of DMF. Additionally, the response values of P30/2 and PA/2 in the NON group were significantly higher than those in the enzyme-treated group (*p* < 0.05), suggesting that enzymatic treatment may reduce the content of irritating and unpleasant odors such as ammonia, amines, hydrogen sulfide, or aldehydes.

Sensors in the T series, including T30/1 (polar organic compounds, hydrogen sulfide), T40/2 (chlorides, fluorides), T70/2 (toluene, xylene), and LY2/LG (chlorides, fluorides, nitrogen oxides, sulfides), exhibited relatively low response values. However, the signal intensity of different groups was still different, which was speculated to be due to the different types and contents of odor precursors produced in different enzymatic processes. Moreover, no significant differences were found in the response values of LY2/LG, indicating that the content of nitrogen oxides (e.g., furans) did not vary substantially and the sensor could not reflect the content of nitrogen–hydrogen compounds such as pyrazines—another class of Maillard reaction products [18].

Figure 2B presents the PCA score plot of response values for the different samples, with PC1 and PC2 accounting for a cumulative variance of 80.8%, which exceeds 75%. This indicates that the model retains most of the sample information. Notably, after different enzymatic treatments, the 95% confidence ellipses of the samples are well separated, demonstrating significant differences between the samples [5,30]. These results confirm that the E-nose can effectively and significantly distinguish differences between the different samples.

In summary, the results demonstrate that E-nose analysis can effectively detect significant differences in DMF odor profiles following enzymatic treatment. While this technique exhibits high sensitivity to global odor profile variations and is well suited for rapid odor classification, it lacks the capacity to resolve individual chemical components within complex mixtures. To comprehensively evaluate whether an enzymatic treatment enhances the flavor quality of fish floss and to elucidate the underlying mechanisms of characteristic flavor formation, more detailed analyses are required.

### 3.3. Effects of Different Proteases on VOCs

#### 3.3.1. HS-GC-IMS Analysis

**Figure 3:**
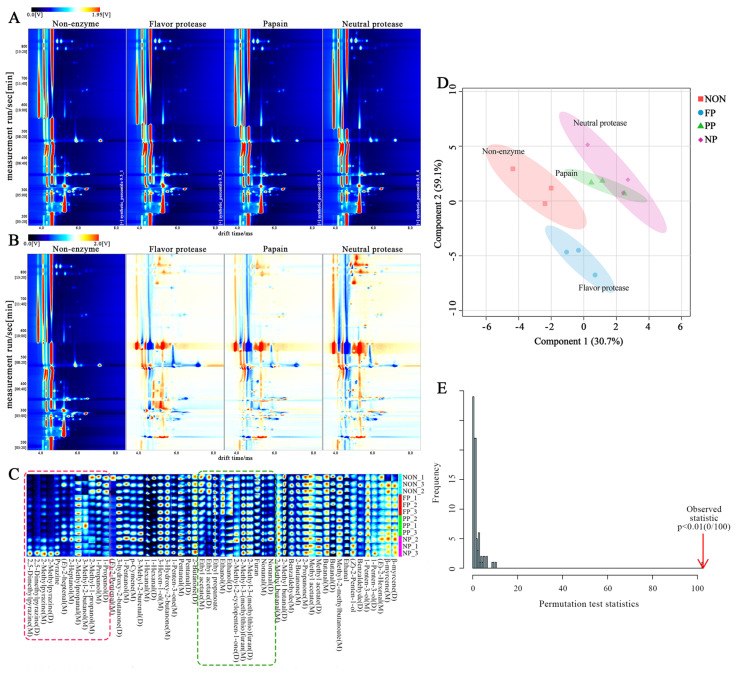
(**A**) Topographic map of GC-IMS spectra of VOCs in DMF treated with different proteases; (**B**) Topographic subtraction map; (**C**) GC-IMS fingerprints; (**D**) PLS-DA score plot of relative contents; (**E**) Permutation test of the PLS-DA model.

HS-GC-IMS was used for the qualitative analysis of VOCs in DMF treated with different proteases. The central red line in Figure 3A is the reactive ion peak (RIP), the bright spots on both sides are the volume peak of each VOC, and the color represents the signal strength of each VOC. The red indicates that the material signal is stronger, and the blue indicates that the material signal is weaker. If the content of VOCs is too high, high-content single compounds and neutral molecules may form a bond in the drift region, generating multiple signals, so that the same compound can simultaneously detect more than one signal, namely monomer and dimer [31]. Furthermore, dimers have a larger relative molecular mass, so the drift time is longer and the distance from RIP is farther [32]. Based on the aforementioned references of GC-IMS, a total of 54 identifiable monomers and dimers are listed in Table 4. The fingerprints of VOCs can be drawn and integrated to obtain results as shown in Figure 3C. Compounds with similar chemical structures can be effectively distinguished through the fingerprints [13]. It can be seen from the red box that the contents of 2,5-dimethylpyrazine, 2-methylpyrazine, pyrazine, (*Z*)-2-heptenal, 2-heptanol, 2-methylpropanal, and other flavor substances in the enzyme-treated group are significantly higher than those in the non-enzyme group, while the contents of 1-propanol and 2-methyl-1-butanol are significantly lower. The contents of compounds such as ethyl acetate, ethyl propanoate, and ethanol in the green box show relatively obvious changes. Figure 3D shows PLS-DA scores of different samples, where the cumulative variance of component 1 and component 2 is 89.8%, greater than 75%, indicating that the model retains most of the information of the samples. Combined with Figure 3B,C, it can be seen that the processed samples can be well distinguished [5,30]. Among them, PP and NP scores have better profiles, and the differences are significant. The PLS-DA model was verified by a permutation test, and random arrangement calculation was adopted [33]. After 100 permutation fittings, Figure 3E shows that *p* < 0.01, indicating that no random grouping model performed better than this model, and the model fitting effect was satisfactory.

#### 3.3.2. Key Volatile Compounds Analysis

**Figure 4:**
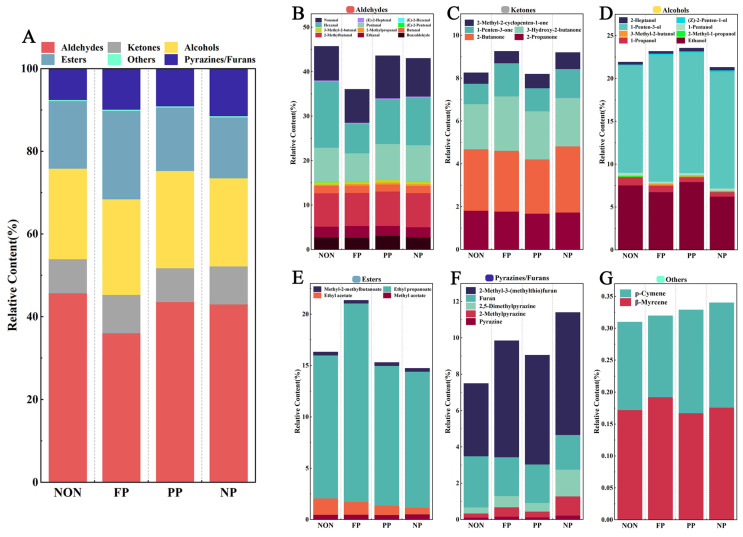
Changes in relative contents of VOCs in DMF treated with different proteases: (**A**) Total VOCs; (**B**) Aldehydes; (**C**) Ketones; (**D**) Alcohols; (**E**) Esters; (**F**) Pyrazines and furans; (**G**) Other VOCs.

The volume peak of each substance was derived for quantitative analysis, and a total of 36 identifiable VOCs were obtained, including 12 aldehydes, 5 ketones, 8 alcohols, 4 esters, and 7 other compounds. Figure 4A (Table S2) shows the relative contents of these substances. We observe that aldehydes had the highest relative content in all groups. Compared with the NON group, the contents of ketones, alcohols, and other compounds remained basically unchanged. In addition, ester substances increased significantly in the FP group, and slightly decreased in the NP group, while the contents of pyrazines and furans increased in all groups. These results are basically consistent with those of an E-nose.

To comprehensively characterize the flavor contribution of VOCs, the calculated relative odor activity value (ROAV) is shown in Table S3, and the relative contents of aldehydes are shown in Figure 4B. The aldehydes in DMF mainly originate from the oxidation of unsaturated fatty acids and Strecker degradation of amino acids [30], exhibiting high relative content in samples. Due to the low olfactory thresholds of aldehydes, the ROAV of 2-methylbutyraldehyde, pentanal, butanal, nonanal, etc., are all greater than 1, providing the main odor contribution across all sample groups; moreover, the detected aldehydes have relatively short carbon chains (C2–C8), mainly imparting grassy and fruity aromas. Additionally, hexanal has a grassy odor at low concentrations and a fishy odor at high concentrations. It is one of the main substances that causes a fishy taste in fish [16]. After enzyme treatment, the relative content of hexanal decreased the most in the FP group, and the relative content was 6.72% (*p* < 0.05). Therefore, FP has a significant de-fishy effect, suggesting its efficacy in enhancing the overall odor profile.

Figure 4C shows the relative contents of ketones. The ketones in DMF mainly originate from unsaturated fatty acid oxidation, amino acid degradation, and MR [29], presenting fruit and cream aromas [30]. Figure 4D shows the relative contents of alcohols. These compounds exhibit relatively high odor thresholds, imparting fruity and floral notes at low concentrations but often generating undesirable pungent odors at high concentrations. However, the ROAV in the samples was all < 1. These results show that the two kinds of substances made little contribution to the flavor, the relative content difference was not significant, and their profiles were not significantly affected by protease addition, suggesting its limited efficacy in enhancing the overall odor profile.

Figure 4E shows the relative contents of esters. Esters present fresh fruity aroma, with ethyl acetate and ethyl propanoate exhibiting relatively high ROAV. Due to the increase in ethyl propanoate, which reached a relative content of 19.37% (*p* < 0.05), indicating enhanced fruity aroma, the ester content of the FP group was the highest after protease treatment. Possibly due to the specificity of FP, more polypeptide bonds of proteins are destroyed, resulting in the loose structure of fish myofibrillar protein; moreover, more lipids are exposed and oxidized and more free fatty acids are produced, thus promoting esterification between fatty acids and alcohols [34]. Despite these changes in ester composition, although esters did not contribute distinct aromatic characteristics to the fish floss or significantly enhance the DMF odor profile, the observed increase in relative content of esters suggests modifications in flavor formation pathways.

Figure 4F shows the relative contents of pyrazines and furans. These compounds contribute nutty, grassy, and roasted meat aromas. Notably, 2,5-dimethylpyrazine exhibited ROAV consistently > 1 across all groups, with enhanced ROAV being observed in protease-treated samples. The NP group exhibited the highest relative contents of pyrazine (0.23%), 2-methylpyrazine (1.05%), 2,5-dimethylpyrazine (1.48%), and 2-methyl-3-(methylthio)furan (6.74%) (*p* < 0.05). These compounds add more unique flavors, such as cooked meat and nut flavors, to DMF [16], demonstrating their significant role in enhancing the DMF odor profile. The increased formation of these substances likely stems from enhanced protein hydrolysis efficiency during enzymatic treatment, which promotes aldol condensation between more FAAs and furfural/Strecker aldehydes [18]. Lastly, the relative contents of the others are shown in Figure 4G. Since this category only consists of two VOCs with low relative contents and no significant differences, their contribution can be considered negligible.

### 3.4. Effects of Different Proteases on FAAs

The present study focused specifically on FAAs as the primary flavor compounds for investigation, based on the following considerations: (1) Although other taste-active components (including free nucleotides, organic acids, inorganic salt ions, etc.) [35] contribute to flavor perception, they remain unaffected by protease treatment and were, therefore, excluded from analysis; (2) While umami peptides represent potential flavor enhancers, their post-hydrolysis diversity and ill-defined threshold values precluded systematic evaluation [36]; (3) As final products of protease hydrolysis, FAAs demonstrate pronounced quantitative changes following enzymatic treatment and represent the greatest contributors to taste profiles [37], making them the most functionally relevant targets for this investigation.

Amino acids exhibit distinct taste attributes, categorized as follows: umami amino acids (UAAs): aspartic acid (Asp) and glutamic acid (Glu); sweet amino acids (SAAs): threonine (Thr), serine (Ser), glycine (Gly), alanine (Ala), and proline (Pro); and bitter amino acids (BAAs): valine (Val), methionine (Met), isoleucine (Ile), leucine (Leu), tyrosine (Tyr), phenylalanine (Phe), lysine (Lys), histidine (His), and arginine (Arg) [28]. To enhance the DMF taste profile, it is essential to promote the content of UAAs and SAAs while either reducing BAAs content or ensuring that their increase occurs at a slower rate compared to that of UAAs and SAAs.

**Figure 5:**
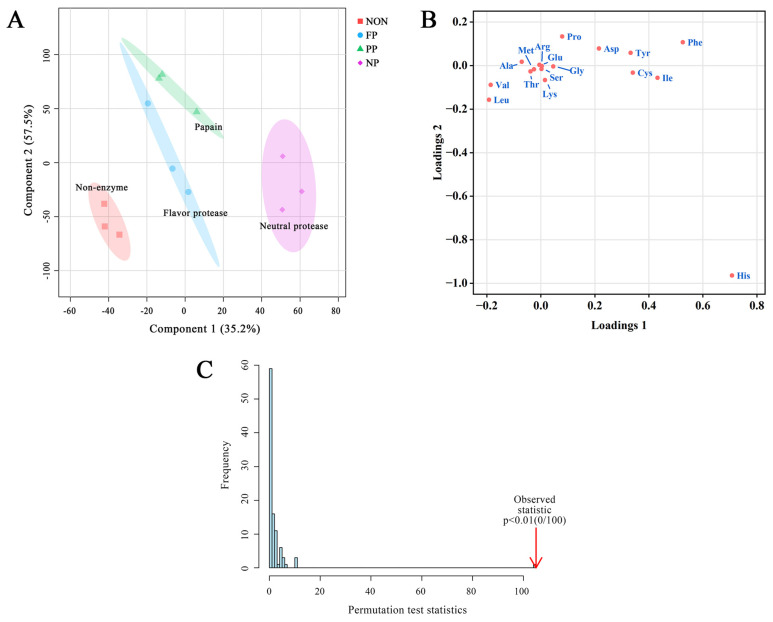
(**A**) PLS-DA score plot of FAA content in DMF treated with different proteases; (**B**) PLS-DA loading plot of FAA content; (**C**) Permutation test of the PLS-DA model.

As shown in the PLS-DA score plot (Figure 5A), the cumulative variance of components 1 and component 2 reached as high as 92.7%, which is greater than 75%, indicating that the model retains most of the information of the samples, and the samples can be well distinguished [5,30]. Among them, the PP group exhibited higher scores along component 2, the NP group dominated component 1, and the non-enzyme group gathered in the third quadrant with lower scores. This spatial separation indicates significant differences in FAA composition between enzyme-treated and non-enzyme-treated groups, demonstrating that the enzymatic hydrolysis process plays a key role in amino acid liberation and taste.

The loading plot (Figure 5B, Table S5) presents distinct spatial distributions of amino acids, as follows:, Pro, Asp, Tyr, and Phe are located in the first quadrant; Val and Leu are located in the third quadrant; Cys and Ile are located in the fourth quadrant near the coordinate axis; His is largely deviated from the other points in the fourth quadrant; while other points are concentrated at the origin of coordinates. Combined with Figure 5A and Table S4, it can be seen that Val and Leu (BAAs) showed positive correlations with the non-enzyme group, serving as effective discriminators for enzyme-treated groups. The FP group gradually approached the origin, with the number of positive correlation types progressively increasing. The positive correlation degree exhibited a rise with UAA (Glu) and SAAs (Gly, Ala), whereas it declined with Val and Leu, ultimately contributing to an enhancement in the DMF taste profile.

The PP group exhibited the closest to the origin in the PLS-DA plot, while Asp, Gly, and Pro were positively correlated with PP as amino acids of umami and sweet taste, respectively, indicating that the PP group not only had the richest flavor profile, but also the most active flavor characteristics. This conclusion can be further supported by taste scores of sensory evaluation shown in Table 3. In contrast, the NP group was primarily located in the fourth quadrant, displaying stronger correlations with BAAs (Tyr, Phe, Cys, Ile, His), Asp and Pro, which were significantly different from the other two groups. The PLS-DA model was verified by a permutation test, random arrangement calculation was adopted [33], and after 100 permutation fittings, Figure 3E shows that *p* < 0.01, indicating that no random grouping model had better results than this model, and the model fitting effect was good.

As shown in Table 5 and Table 6, protease treatment significantly increased the liberation of FAAs that contribute to flavor enhancement in DMF. Previous studies have demonstrated that different sources and types of proteases have different effects on the structure of myofibrillar proteins, primarily attributed to variations in the characteristics of the active site of enzyme-activated proteins and the mechanism of the formation of flavor substances [38,39,40]. Consequently, both the content and type of hydrolyzed amino acids showed different variations. In terms of content, the amino acid content of both PP and NP groups increased compared with that of the NON group. Regarding types, the total increase in UAAs and SAAs was higher than that of BAAs. Specifically, the PP treatment induced 26.68% and 25.98% increments in UAAs and SAAs, respectively, which was higher than that of BAAs (11.99%), while the increase in UAAs in the NP group was 34.96% and 15.84%, respectively, which was higher than that of BAAs (23.35%). These results suggest that both PP and NP could positively enhance the flavor of DMF, with PP demonstrating the most pronounced enhancement. Conversely, the negative increase in UAAs and BAAs and the slight increase in SAAs in the FP group may be due to the different binding sites of FPs, which produce more peptides than FAAs, or due to targeted cleavage of specific peptide bonds, which exposes more fats to FAAs in an MR that generates unidentified flavor substances.

**Figure 6:**
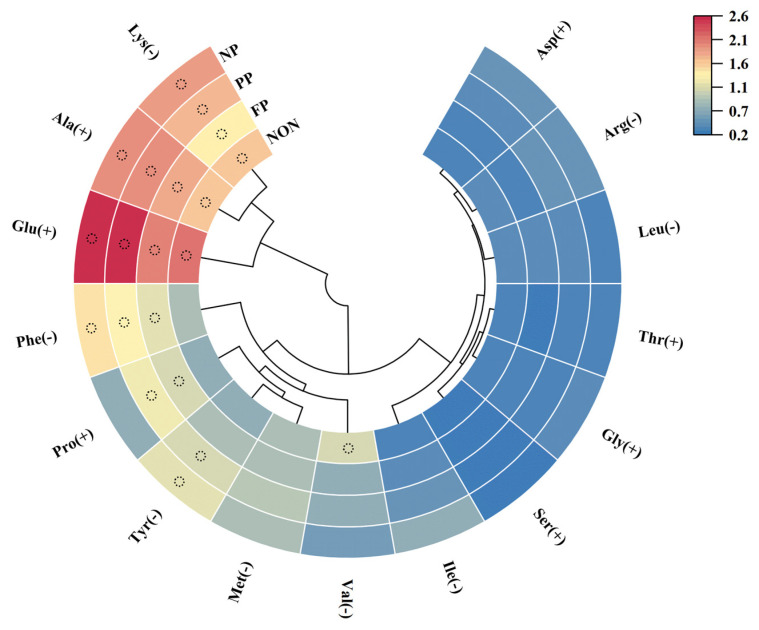
Cluster heatmap of TAV for FAAs in DMF treated with different proteases (excluding His (−), the black circles in the figure are the substances with TAV > 1).

To further analyze the taste characteristics of FAAs in DMF, the key taste substances were screened by calculating taste activity value (TAV), with TAV > 1 indicating primary taste contributors. Moreover, higher TAV of UAAs and SAAs were found to produce more pronounced flavor enhancement effects, as illustrated in Figure 6 (Table S4), and the TAV of the His of four groups can be seen in Table S4. The TAV of Glu (UAA) and Ala (SAA) were all greater than 1. Still, the TAV of the enzyme-treated group were higher, making their freshness and sweetness characteristics more pronounced; moreover, the PP group in particular showed the highest TAV for both Glu (2.61) and Ala (2.04) among all groups. In addition, Pro (SAA) demonstrated TAV > 1 exclusively in the FP and PP groups, establishing its role as a key taste-active component in these treatments. However, the TAV of the PP group was still the highest, at 1.23. Other amino acids exhibited TAV < 1 across all groups, indicating negligible flavor contributions. Regarding BAAs, His consistently exhibited the highest TAV among all four groups. Phe transitioned from TAV < 1 in the non-enzyme group to TAV > 1 in the enzyme-treated group, while Val had TAV > 1 in the non-enzyme group and TAV < 1 in the enzyme-treated group. Cross-referencing with Table 6 suggests no substantial alteration in bitter taste perception. In summary, the TAV analysis of FAAs revealed that PP most significantly enhanced the taste profile of DMF, which is consistent with the results presented in Table 3. Additionally, His can be enzymatically converted to histamine via bacterial decarboxylation, which leads to uncomfortable symptoms such as dizziness and rapid heartbeat in humans [41], whereas the His content (Table S4) in the NP group of enzyme-treated samples was significantly higher than that of the FP and PP groups (*p* < 0.05). This difference likely arises from the stronger proteolytic activity of the NP group, which also exhibited the highest levels of most other FAAs (*p* < 0.05). To reduce His content in the NP group while retaining flavor-enhancing FAAs, optimization approaches may include modifying ingredient formulations and adjusting MR conditions. Further investigation is required to determine the most effective strategies.

### 3.5. Flavor Correlation Analysis

Previous studies have shown that the flavor of fish floss primarily originates from MR and lipid oxidation pathways [5]. For the MR, amino acids not only act as flavor-presenting substances, but also can participate in the MR as precursors for flavor compound synthesis. This process initiates through carbonyl-amine condensation between amino groups and reducing sugar-derived carbonyl/aldehyde groups, progressing sequentially via Amadori rearrangement, Heyns rearrangement, and Strecker degradation, ultimately generating characteristic VOCs [42]. For lipid oxidation, depending on the carbon chain length, unsaturation, and positional isomerism of double bonds of different fatty acids, the substances produced by oxidation are different [43], and they can be broadly categorized as aldehydes, ketones, alcohols, and carboxylic acids. Given the substantial lipid content and high polyunsaturated fatty acid composition characteristic of mackerel, lipid oxidation also contributes significantly to the flavor of fish floss [3,44].

**Figure 7:**
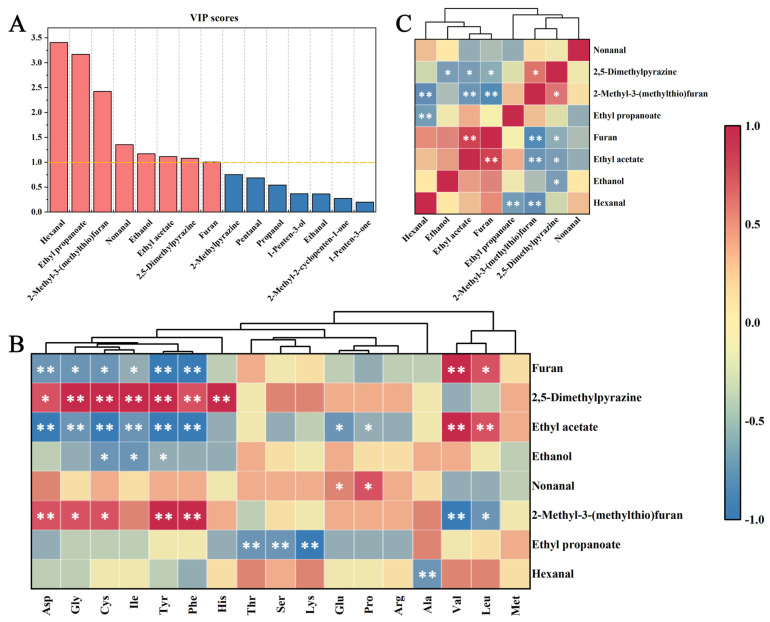
(**A**) VIP scores of VOCs in DMF treated with different proteases. (**B**) Correlation heatmap between FAAs and VOCs. (**C**) Correlation heatmap among VOCs. (* indicates significant correlation (*p* < 0.05), and ** indicates highly significant correlation (*p* < 0.01)).

Due to the diverse range of VOCs detected, a targeted screening of critical substances was implemented to assist in focused correlation analysis. Based on the initial data presented in Figure 3B, the VIP scores of the top 15 VOCs were determined (Figure 7A, Table S6). Compounds with VIP scores > 1 were designated as key VOCs [30]. This analysis identified hexanal, ethyl propanoate, 2-methyl-3-(methythio)furan, nonanal, ethanol, ethyl acetate, 2,5-dimethylpyrazine, and furan as the primary contributors to the flavor of the DMF. These results demonstrate substantial agreement with the ROAV analysis of VOCs.

A correlation heatmap was constructed with FAAs as the x-axis and key VOCs as the y-axis (Figure 7B, Table S7). For a clearer discussion, the VOCs were classified into two groups: hydrocarbons and nitrogen-containing compounds.

For hydrocarbons, ethyl acetate exhibited a positive correlation (*p* < 0.01) with Val and Leu, while showing negative correlations (*p* < 0.01) with Asp, Gly, Cys, Ile, Tyr, and Phe. Negative correlations (*p* < 0.05) were also observed with Glu and Pro. This phenomenon may be attributed to the polar nature of Asp, Cys, Tyr, and Glu, which exhibit limited solubility in ethyl acetate. Given the low moisture content of DMF, these FAAs may have been predominantly consumed through MR due to exposure to air. Conversely, nonpolar amino acids (Gly, Ile, Phe, Val, Leu, and Pro) demonstrate solubility in lipids and ethyl acetate. During the stir-frying process, the interplay between MR and lipid oxidation generates complex interactions [5]. Partial lipids remained intact, while others underwent oxidation to generate aldehydes, ketones, and acids. These degradation products subsequently underwent dehydration–condensation under high-temperature conditions to form esters, suggesting that reduced lipid availability may enhance ester formation through this pathway. Due to the inherent diversity and structural complexity of the intermediate products, there are uncertainties in the overall properties of the dynamic intermolecular interactions, resulting in a positive correlation between Val and Leu being more soluble in esters, and a negative correlation between Gly, Ile, Phe, and Pro being more soluble in other nonpolar substances. The specific reasons need to be further investigated.

Nonanal, a characteristic VOC of mackerel lipid oxidation [45], showed positive correlations with Glu and Pro (*p* < 0.05), but negative correlations with Val and Leu. This observation suggests potential solubility preferences of Pro and Glu in nonanal. Hexanal exhibited a negative correlation with Ala (*p* < 0.01), but a positive correlation with ethyl propanoate, suggesting preferential solubility of Ala in ethyl propanoate. These correlation patterns between nonanal and hexanal corroborate the proposed ethyl acetate interaction mechanisms. Additional observations include ethanol’s negative correlations with Cys, Tyr, and Ile (*p* < 0.05), and ethyl propanoate’s negative correlations with Thr, Ser, and Lys (*p* < 0.01). These findings align with the correlation patterns observed in the ethyl acetate analysis.

For nitrogen-containing compounds, FAAs serve as essential reactants in MR, undergoing complex transformations, including Amadori rearrangement, Heyns rearrangement, and Strecker degradation, to yield low-molecular-weight nitrogen-containing compounds [42]. In this study, these compounds primarily included flavor-active substances such as furan, 2,5-dimethylpyrazine, and 2-methyl-3-(methylthio)furan. Furan demonstrated negative correlations (*p* < 0.05) with Asp, Tyr, Phe, Gly, Cys, and Ile. Conversely, 2,5-dimethylpyrazine and 2-methyl-3-(methylthio)furan exhibited positive correlations (*p* < 0.05, except for the 2-methyl-3-(methylthio)furan-Ile interaction) with these amino acids. These findings suggest competitive pathways in nitrogen-containing formation during MR, where specific amino acids suppress furan generation while promoting the production of pyrazine and thioether derivatives.

Notably, furan demonstrated positive correlations with Val and Leu (*p* < 0.05), contrasting with the negative correlations (*p* < 0.05) observed between 2-methyl-3-(methylthio)furan and these branched-chain amino acids. Such divergence implies that Val and Leu preferentially participate in reaction pathways favoring furan synthesis over thioether compound formation. His, the most abundant BAA with the highest taste activity value (TAV) in DMF, exhibited a strong positive correlation with 2,5-dimethylpyrazine (*p* < 0.01). It can be inferred that the MR can consume part of His to produce new VOCs, as the enzyme-treated group produced His faster than the consumption rate, and thus showed an increasing trend of His content.

Based on the correlation analysis of hydrocarbons with nitrogen-containing compounds (Figure 7C, Table S7), and taking into account the previous finding that enzymatic hydrolysis of prawn myofibrillar protein promotes the formation of pyrazines [46], the main pathways and key control points for the formation of VOCs through lipid oxidation and MR can be inferred. There was a positive correlation between ethyl acetate and furan derivatives (*p* < 0.01). This correlation results from the accumulation of ethyl acetate through the dehydrated condensation of acetic acid and ethanol derived from lipid oxidation. The enhanced solubility of Val and Leu in ethyl acetate helps to improve their availability as furan precursors. Subsequent complex reactions, including Amadori rearrangement, Heyns rearrangement, and Strecker degradation, under specific conditions drive furan formation. The formation pathways and key mechanisms of other compounds follow similar principles: (1) Val and Leu serve as precursors for furan formation. Asp, Tyr, Phe, Gly, Cys, and Ile act as precursors for 2,5-dimethylpyrazine and 2-methyl-3-(methylthio)furan. (2) Ethyl acetate indirectly suppresses the formation of 2-methyl-3-(methylthio)furan, and ethanol indirectly suppresses the formation of 2,5-dimethylpyrazine. Furthermore, competitive interactions were observed among key VOCs: 2,5-dimethylpyrazine exhibited mutual inhibition with 2-methyl-3-(methylthio)furan, and furan also exhibited mutual inhibition with both 2-methyl-3-(methylthio)furan and 2,5-dimethylpyrazine.

## 4. Conclusions

This study demonstrated that FP and NP exhibited the most significant enhancement in the odor characteristics of DMF, primarily due to a reduction in hexanal (fishy odor) content and an increase in nitrogen-containing compounds (nutty aroma). In contrast, PP showed optimal effectiveness in enhancing the taste profile, which was attributed to elevated levels of SAAs and UAAs combined with negligible changes in BAAs. Further analysis identified Val and Leu as precursors for furan derivatives, with concurrent suppression of 2-methyl-3-(methylthio)furan formation. Additionally, Asp, Tyr, Phe, Gly, Cys, and Ile were established as precursors for both 2,5-dimethylpyrazine and 2-methyl-3-(methylthio)furan, while inhibiting furan formation. In summary, minimal enzyme supplementation at 10 U/g effectively enhanced flavor profiles in DMF. Moreover, a novel pathway for the formation of nitrogen-containing flavor-enhancing compounds was elucidated. Additionally, this study establishes a theoretical framework for the enzymatic processing of fish meat using protease complexes.

## Figures and Tables

**Table 1:** Sensory assessment of dried mackerel floss.

Index	Weight	Standard	Score Range
Texture	10%	Fluffy fibers without agglomeration	81~100
		Fluffy fibers with minor agglomeration	61~80
		Indistinct fluffiness, compact fibers with agglomeration	0~60
Color	20%	Uniform and appealing coloration	81~100
		Moderate coloration with acceptable uniformity	61~80
		Poor coloration with partial charring and non-uniformity	0~60
Odor	35%	Pure aroma without off-flavors or fishy notes	81~100
		Mild aroma with slight fishiness	61~80
		Faint aroma with pronounced fishy odors	0~60
Taste	35%	Well-balanced sweetness and saltiness with umami, no bitterness	81~100
		Balanced sweetness/saltiness with partial umami and slight bitterness	61~80
		Overly sweet/salty, lacking umami, with noticeable bitterness	0~60

**Table 2:** Sensors and corresponding responsive substances of the E-nose.

NO.	Sensors	Responsive Substances
1	LY2/LG	chloride, fluorine, nitrogen oxide, sulfide
2	LY2/G	ammonia, amines, carbon oxide
3	LY2/AA	ammonia, ethanol, propanone
4	LY2/GH	ammonia, amines
5	LY2/gCTL	hydrogen sulfide
6	LY2/gCT	propane, butane
7	P10/1	carbon oxide, ammonia, chlorine
8	P10/2	methane, ethane
9	P30/1	carbon oxide, ethanol, hydrocarbon, ammonia
10	P30/2	aldehydes, ethanol, hydrocarbon, hydrogen sulfide
11	P40/1	chloride, fluoride
12	P40/2	hydrogen sulfide, chlorine, fluoride
13	PA/2	ethanol, ammonia, amines
14	T30/1	polar organic compounds, hydrogen sulfide
15	T40/1	fluoride
16	T40/2	chloride, fluoride
17	T70/2	toluene, xylene
18	TA/2	ethanol

**Table 3:** Sensory scores of DMF treated with different proteases.

Group	Texture	Color	Odor	Taste	Total Score
NON	81.30 ± 6.48 ^a^	77.80 ± 7.11 ^b^	75.20 ± 8.75 ^b^	79.30 ± 5.52 ^b^	78.12 ± 5.24 ^c^
FP	84.60 ± 6.33 ^a^	77.70 ± 4.32 ^b^	85.40 ± 4.25 ^a^	84.10 ± 4.18 ^b^	84.05 ± 2.07 ^ab^
PP	80.50 ± 6.04 ^a^	82.60 ± 5.30 ^ab^	81.50 ± 3.57 ^a^	91.40 ± 5.48 ^a^	84.88 ± 1.96 ^a^
NP	78.40 ± 7.21 ^a^	84.30 ± 6.41 ^a^	83.30 ± 7.21 ^a^	79.70 ± 6.78 ^b^	81.16 ± 4.74 ^bc^

Note: Different lowercase letters within the same row indicate significant differences (*p* < 0.05).

**Table 4:** Integration parameters of VOCs identified by GC-IMS.

Compounds	CAS#	Formula	RI	Rt [sec]	Dt [a.u.]
**Aldehydes(19)**					
Benzaldehyde(M)	C100527	C_7_H_6_O	1531.8	1400.889	1.15497
Benzaldehyde(D)	C100527	C_7_H_6_O	1519.2	1361.248	1.4536
Ethanal(M)	C75070	C_2_H_4_O	754.9	215.283	0.95755
2-Methylbutanal(M)	C96173	C_5_H_10_O	914.7	303.97	1.14825
2-Methylbutanal(D)	C96173	C_5_H_10_O	920.6	307.873	1.39969
Butanal(M)	C123728	C_4_H_8_O	834.2	255.456	1.11441
Butanal(D)	C123728	C_4_H_8_O	825.7	250.857	1.28115
2-Methylpropanal(M)	C78842	C_4_H_8_O	769.8	222.342	1.09414
3-Methyl-2-butenal(M)	C107868	C_5_H_8_O	1189	656.316	1.35782
3-Methyl-2-butenal(D)	C107868	C_5_H_9_O	1189.6	657.711	1.35916
(*E*)-2-Pentenal(M)	C1576870	C_5_H_8_O	1142	563.256	1.10319
Pentanal(M)	C110623	C_5_H_10_O	987.6	355.774	1.17861
Pentanal(D)	C110623	C_5_H_10_O	994.4	361.081	1.42165
Hexanal(M)	C66251	C_6_H_12_O	1103.1	496.084	1.27839
Hexanal(D)	C66251	C_6_H_12_O	1092.7	480.388	1.56221
(*E*)-2-Hexenal(M)	C6728263	C_6_H_10_O	1224.8	708.85	1.18641
(*E*)-2-Heptenal(M)	C6728263	C_6_H_11_O	1324.1	873.941	1.26129
Nonanal(M)	C124196	C_9_H_18_O	1398.7	1035.318	1.47503
Nonanal(D)	C124196	C_9_H_18_O	1396.3	1029.702	1.94061
**Ketones(8)**					
2-Propanone(M)	C67641	C_3_H_6_O	834.2	255.494	1.11326
2-Butanone(M)	C78933	C_4_H_8_O	908.9	300.197	1.05918
2-Butanone(D)	C78933	C_4_H_8_O	906.6	298.713	1.24886
3-Hydroxy-2-butanone(M)	C513860	C_4_H_8_O_2_	1288.3	806.628	1.06282
3-Hydroxy-2-butanone(D)	C513860	C_4_H_8_O_2_	1288.6	807.132	1.33028
1-Penten-3-one(M)	C1629589	C_5_H_8_O	1020.8	389.072	1.07945
2-Methyl-2-cyclopenten-1-one(M)	C1120736	C_6_H_8_O	1338.9	903.708	1.11959
2-Methyl-2-cyclopenten-1-one(D)	C1120736	C_6_H_8_O	1334.9	895.703	1.43103
**Alcohols(10)**					
Ethanol(M)	C64175	C_2_H_6_O	957.1	333.103	1.04201
Ethanol(D)	C64175	C_2_H_6_O	944.6	324.245	1.12393
1-Propanol(M)	C71238	C_3_H_8_O	1046.6	419.637	1.11297
1-Propanol(D)	C71238	C_3_H_8_O	1047.2	420.383	1.24984
2-Methyl-1-propanol(M)	C78831	C_4_H_10_O	1102.4	494.965	1.17207
3-Methyl-2-butanol(M)	C598754	C_5_H_12_O	1136.6	553.331	1.23485
1-Pentanol(M)	C71410	C_5_H_12_O	1256.8	756.431	1.25639
1-Penten-3-ol(M)	C616251	C_5_H_10_O	1158.9	595.132	0.94746
(*Z*)-2-Penten-1-ol(M)	C1576950	C_5_H_10_O	741.9	209.318	0.9434
2-Heptanol(M)	C543497	C_7_H_16_O	1323.2	872.026	1.3838
**Esters(6)**					
Methyl acetate(M)	C79209	C_3_H_6_O_2_	844.2	261.059	1.0285
Methyl acetate(D)	C79209	C_3_H_6_O_2_	842.9	260.317	1.19236
Ethyl acetate(M)	C141786	C_4_H_8_O_2_	890.5	288.511	1.09792
Ethyl acetate(D)	C141786	C_4_H_8_O_2_	890.2	288.326	1.33604
Ethyl propanoate(M)	C105373	C_5_H_10_O_2_	937.6	319.372	1.15107
Methyl-2-methylbutanoate(M)	C868575	C_6_H_12_O_2_	1029.7	399.332	1.18999
**Pyrazines/Furans(8)**					
Pyrazine(M)	C290379	C_4_H_4_N_2_	1217.5	698.283	1.05229
2-Methylpyrazine(M)	C109080	C_5_H_6_N_2_	1268.7	774.968	1.09096
2-Methylpyrazine(D)	C109080	C_5_H_6_N_2_	1268.3	774.477	1.39484
2,5-Dimethylpyrazine(M)	C123320	C_6_H_8_N_2_	1319.8	865.458	1.11654
2,5-Dimethylpyrazine(D)	C123320	C_6_H_8_N_2_	1318.3	862.538	1.49925
Furan(M)	C110009	C_4_H_4_O	782.3	228.413	0.94456
2-Methy-3-(methylthio)furan(M)	C63012975	C_6_H_8_OS	1304.1	835.171	1.10871
2-Methyl-3-(methylthio)furan(D)	C63012975	C_6_H_8_OS	1302.8	832.573	1.15225
**Others(3)**					
β-Myrcene(M)	C123353	C_10_H_16_	1142.3	563.684	1.28537
β-Myrcene(D)	C123353	C_10_H_16_	1142.1	563.388	1.22133
p-Cymene(M)	C99876	C_10_H_14_	1225.1	709.288	1.30361

Note: RI: retention index; RT: retention time; DT: drift time; (M): monomer; (D): dimer.

**Table 5:** Content of FAA varieties in DMF treated with different proteases.

	Content(mg/100 g)		
	UAA	SAA	BAA
NON	93.23	259.97	1378.25
FP	87.70	273.84	1352.94
PP	118.11	327.26	1543.49
NP	125.82	301.14	1700.12

**Table 6:** Relative increase in the amplitude of FAAs in DMF treated with different proteases compared to NON.

	Increase in Content Relative to NON(%)		
	UAA	SAA	BAA
FP	−5.94	5.34	−1.84
PP	26.68	25.89	11.99
NP	34.96	15.84	23.35

## Data Availability

The original contributions presented in the study are included in the article/Supplementary Materials, further inquiries can be directed to the corresponding authors.

## Supplementary Materials

The following supporting information can be downloaded at: https://www.mdpi.com/article/10.3390/foods14111864/s1, Table S1: E-nose sensor response value of DMF treated with different proteases; Table S2: Relative content of VOCs in DMF treated with different proteases; Table S3: ROAV of some VOCs in DMF treated with different proteases; Table S4: FAA content and (TAV) in DMF treated with different proteases; Table S5: PLS-DA loadings of FAAs in DMF; Table S6: VIP values of VOCs in DMF; Table S7: Correlation coefficients between FAAs and VOCs in DMF.

## Abbreviations

The following abbreviations are used in this manuscript:

BAA	Bitter amino acid
DMF	Dried mackerel floss
E-nose	Electronic nose
FAA	Free amino acid
FP	Flavor protease
MR	Maillard reaction
NON	Non-enzyme
NP	Neutral protease
PCA	Principal components analysis
PLS-DA	Partial least squares discrimination analysis
PP	Papain
ROAV	Relative odor activity value
SAA	Sweet amino acid
TAV	Taste activity value
UAA	Umami amino acid
VIP	Variable importance in projection
VOC	Volatile organic compound
