# Supplementary material for: Enhancing Flavor in Dried Mackerel Floss (Scomberomorus niphonius) via Protease: Formation Mechanism of Characteristic Flavor Revealed by Integrated Multi-Omics Analysis
Source: Foods. 2025 May 24;14(11):1864. doi: 10.3390/foods14111864 (PMC12155409; doi:10.3390/foods14111864)
Supplement: Supplementary file 1 [file foods-14-01864-s001.zip › foods-3634091-supplementary.pdf]

Table S1. E-nose sensor response value of DMF treated with different proteases

Senors	Response value			
	NON	FP	PP	NP
LY2/LG	0.5423±0.0254[a]	0.5718±0.0174[a]	0.5581±0.0085[a]	0.5428±0.0109[a]
LY2/G	-0.1389±0.0123[b]	-0.1068±0.0061[a]	-0.1620±0.0059[c]	-0.1228±0.0124[ab]
LY2/AA	-0.1265±0.0028[b]	-0.0920±0.0052[a]	-0.1309±0.0044[b]	-0.1017±0.0081[a]
LY2/GH	-0.1727±0.0103[b]	-0.1288±0.0049[a]	-0.2034±0.0077[c]	-0.1540±0.0154[b]
LY2/gCTL	-0.1474±0.0088[b]	-0.1320±0.0067[a]	-0.1623±0.0021[c]	-0.1378±0.0073[ab]
LY2/gCT	-0.0980±0.0051[a]	-0.1029±0.0076[a]	-0.1070±0.0020[a]	-0.0990±0.0040[a]
P10/1	0.4271±0.0116[a]	0.4145±0.0281[a]	0.4063±0.0147[a]	0.3930±0.0069[a]
P10/2	0.4846±0.0167[ab]	0.4631±0.0231[b]	0.4959±0.123[a]	0.4745±0.0053[ab]
P30/1	0.5055±0.0045[a]	0.5093±0.0252[a]	0.4873±0.0113[ab]	0.4642±0.0150[b]
P30/2	0.7775±0.0117[a]	0.7578±0.0193[ab]	0.7512±0.0063[b]	0.7216±0.0086[c]
P40/1	0.3879±0.0167[a]	0.3714±0.0253[a]	0.3728±0.0143[a]	0.3620±0.0080[a]
P40/2	0.3668±0.0025[b]	0.3627±0.0133[b]	0.3873±0.0062[a]	0.3657±0.0073[b]
PA/2	0.7733±0.0150[a]	0.7316±0.0209[b]	0.7599±0.0072[a]	0.7293±0.0035[b]
T30/1	0.5453±0.0040[b]	0.5305±0.0146[b]	0.5740±0.0073[a]	0.5426±0.0052[b]
T40/1	0.2124±0.0020[a]	0.2157±0.0185[a]	0.2263±0.0096[a]	0.2169±0.0064[a]
T40/2	0.4935±0.0034[ab]	0.4914±0.0177[ab]	0.5067±0.0081[a]	0.4840±0.0086[b]
T70/2	0.4419±0.0076[ab]	0.4234±0.0148[b]	0.4568±0.0076[a]	0.4288±0.0055[b]
TA/2	0.2367±0.0008[a]	0.2397±0.0208[a]	0.2518±0.0108[a]	0.2435±0.0099[a]

Note: Different lowercase letters within the same row indicate significant differences ($p < 0.05$).

Table S2. Relative content of VOCs in DMF treated with different proteases

Compound	Relative Content%			
	Non-enzyme	Flavor protease	Papain	Neutral protease
**Aldehydes(12)**				
Benzaldehyde	2.77±0.29[ab]	2.67±0.04[ab]	3.10±0.13[a]	2.77±0.18[b]
Ethanal	2.42±0.09[ab]	2.66±0.36[a]	2.23±0.03[b]	2.28±0.14[ab]
2-Methylbutanal	7.49±0.12[a]	7.43±0.33[a]	7.75±0.43[a]	7.67±0.74[a]
Butanal	1.72±0.04[a]	1.67±0.17[a]	1.57±0.12[a]	1.62±0.21[a]
2-Methylpropanal	0.22±0.04[b]	0.40±0.03[a]	0.43±0.03[a]	0.40±0.03[a]
3-Methyl-2-butenal	0.52±0.03a[b]	0.40±0.05[b]	0.64±0.11[a]	0.58±0.13a[b]
(*E*)-2-Pentenal	0.07±0.08[a]	0.03±0.00[a]	0.03±0.00[a]	0.02±0.00[a]
Pentanal	7.74±0.28[a]	6.42±0.24[b]	8.01±0.35[a]	8.14±0.26[a]
Hexanal	14.90±0.01[a]	6.72±2.08[b]	10.05±1.85[ab]	10.77±3.45[ab]
(*E*)-2-Hexenal	0.06±0.01[a]	0.05±0.00[a]	0.05±0.00[ab]	0.04±0.00[b]
(*E*)-2-Heptenal	0.17±0.07[a]	0.11±0.01[a]	0.18±0.04[a]	0.16±0.11[a]
Nonanal	7.65±0.18[b]	7.49±0.91[b]	9.55±0.55[a]	8.57±1.47[ab]
**Total**	45.71	36.05	43.6	43.02
**Ketones(5)**				
2-Propanone	1.82±0.03[a]	1.78±0.17[a]	1.68±0.12[a]	1.74±0.20[a]
2-Butanone	2.87±0.11[ab]	2.84±0.18[ab]	2.53±0.10[b]	3.09±0.40[a]
3-Hydroxy-2-butanone	2.10±0.06[b]	2.54±0.07[a]	2.24±0.09[b]	2.26±0.21[b]
1-Penten-3-one	0.95±0.03[c]	1.55±0.11[a]	1.08±0.05[c]	1.35±0.14[b]
2-Methyl-2-cyclopenten-1-one	0.51±0.06[b]	0.56±0.03[b]	0.66±0.03[ab]	0.77±0.07[a]
**Total**	8.25	9.26	8.19	9.2
**Alcohols(8)**				
Ethanol	7.54±0.37[a]	6.75±0.33[b]	7.92±0.07[a]	6.23±0.54[b]
1-Propanol	0.97±0.03[a]	0.76±0.04[b]	0.58±0.02[c]	0.53±0.05[c]
2-Methyl-1-propanol	0.10±0.02[a]	0.06±0.00[b]	0.05±0.01[b]	0.05±0.01[b]
3-Methyl-2-butanol	0.07±0.04[c]	0.19±0.07[a]	0.15±0.00[ab]	0.10±0.02[bc]
1-Pentanol	0.33±0.05[a]	0.22±0.01[a]	0.28±0.06[a]	0.25±0.11[a]
1-Penten-3-ol	12.49±0.31[b]	14.78±0.23[a]	14.05±0.40[ab]	13.58±1.16[ab]
(*Z*)-2-Penten-1-ol	0.13±0.01[b]	0.16±0.02[b]	0.16±0.01[b]	0.22±0.02[a]
2-Heptanol	0.28±0.06[a]	0.25±0.01[a]	0.36±0.04[a]	0.34±0.10[a]
**Total**	21.92	23.17	23.55	21.31
**Esters(4)**				
Methyl acetate	0.48±0.01[a]	0.48±0.02[ab]	0.46±0.01[b]	0.52±0.06[ab]
Ethyl acetate	1.59±0.12[a]	1.24±0.08[b]	0.91±0.03[c]	0.64±0.04[d]
Ethyl propanoate	13.92±0.86[b]	19.34±1.00[a]	13.60±0.17[b]	13.24±0.86[b]
Methyl-2-methylbutanoate	0.33±0.02[a]	0.30±0.01[a]	0.32±0.02[a]	0.33±0.02[a]
**Total**	16.31	21.36	15.29	14.73
**Pyrazines/Furans(5)**				
Pyrazine	0.12±0.00[b]	0.17±0.01[b]	0.13±0.01[b]	0.23±0.06[a]
2-Methylpyrazine	0.23±0.01[b]	0.51±0.07[b]	0.32±0.03[b]	1.05±0.48[a]
2,5-Dimethylpyrazine	0.33±0.01[b]	0.62±0.09[b]	0.47±0.04[b]	1.48±0.65[a]
Furan	2.82±0.19[a]	2.14±0.10[a]	2.12±0.11[a]	1.91±0.18[a]
2-Methyl-3-(methylthio)furan	4.01±0.50[b]	6.41±0.39[a]	6.01±0.42[a]	6.74±0.46[a]
**Total**	7.5	9.84	9.05	11.4
**Others(2)**				
β-Myrcene	0.17±0.07[a]	0.19±0.02[a]	0.17±0.01[a]	0.18±0.01[a]
p-Cymene	0.14±0.02[a]	0.13±0.01[b]	0.16±0.02[b]	0.16±0.04[b]
**Total**	0.31	0.32	0.33	0.34

Note: Different lowercase letters within the same row indicate significant differences ($p < 0.05$).

Table S3. ROAV of some VOCs in DMF treated with different proteases

Compound	OT (μg/kg)	Odour description	ROAV			
			NON	FP	PP	NP
Benzaldehyde	350	Almond and cherry aroma	0.10	0.10	0.09	0.09
Ethanal	25	Pungent with green apple notes	1.27	1.42	0.94	1.07
2-Methylbutanal	1	Caramel, banana, and apple fragrance	97.97	99.09	81.21	89.47
Butanal	17	Grassy scent	1.32	1.31	0.97	1.11
2-Methylpropanal	4.4	Malt-like aroma	0.66	1.23	1.02	1.05
Pentanal	12	Grassy and fruity notes	8.43	7.14	6.99	7.92
Hexanal	5	Grassy, vegetal, and fruity bouquet	38.97	17.94	21.06	25.15
(E)-2-Heptenal	13	Grassy, cucumber, and vegetal notes	0.17	0.11	0.15	0.14
Nonanal	1	Cured meat, rose, and fresh citrus peel aroma	100.00	100.00	100.00	100.00
2-Butanone	33	Sweet and fruity fragrance	1.14	1.15	0.80	1.09
Ethanol	10000	Sharp with fruity and floral undertones	0.01	0.01	0.01	0.01
2-Methyl-1-propanol	3200	Pungent, sweet yet bitter	0.00	0.00	0.00	0.00
1-Pentanol	4000	Grassy and greasy with a spicy hint	0.00	0.00	0.00	0.00
1-Penten-3-ol	400	Cured meat, mushroom, and earthy odour	0.41	0.49	0.37	0.40
Ethyl acetate	5	Fresh fruity and floral scent	4.16	3.31	1.91	1.49
Ethyl propanoate	10	Refreshing pineapple aroma	18.20	25.80	14.25	15.45
Methyl 2-methylbutanoate	4.4	Apple and tropical fruit notes	0.97	0.91	0.77	0.87
2,5-Dimethylpyrazine	1.8	Nutty, roasted potato, and caramelized tones	2.40	4.61	2.74	9.57

Note: OT: olfactory threshold.

Table S4. FAA content and (TAV) in DMF treated with different proteases

Amino Acid	Flavor Attribute	TT (mg/100g)	Content(mg/100g)				TAV			
			NON	FP	PP	NP	NON	FP	PP	NP
Asp	Unami(+)	100	22.87±3.42^{d}	29.31±1.16^{c}	44.63±0.03^{b}	48.28±1.26^{a}	0.29	0.26	0.40	0.48
Thr	Sweet(+)	260	71.93±0.90^{a}	54.16±11.87^{b}	72.68±2.69^{a}	70.00±1.68^{a}	0.25	0.21	0.28	0.28
Ser	Sweet(+)	150	29.53±1.20^{a}	26.77±0.31^{b}	30.05±0.92^{a}	31.19±1.41^{a}	0.18	0.16	0.20	0.21
Glu	Unami(+)	30	70.82±0.27^{b}	69.58±4.04^{b}	78.24±0.92^{a}	75.26±1.56^{a}	2.14	2.06	2.61	2.58
Gly	Sweet(+)	130	36.71±0.34^{b}	38.70±1.89^{b}	40.93±0.77^{ab}	44.13±3.94^{a}	0.26	0.27	0.31	0.34
Ala	Sweet(+)	60	110.28±1.02^{b}	121.44±5.81^{a}	122.22±1.91^{a}	113.61±3.73^{b}	1.68	1.81	2.04	2.00
Cys	Bitter/Sweet/Sulfurous(-)	ND	41.10±0.68^{d}	46.17±2.03^{c}	46.54±2.24^{b}	75.15±0.41^{a}	-	-	-	-
Val	Bitter/Sweet(-)	40	39.77±0.19^{a}	26.00±1.48^{b}	22.87±2.45^{c}	20.23±1.60^{c}	1.05	0.72	0.68	0.51
Met	Bitter/Sweet/Sulfurous(-)	30	27.03±0.89^{a}	27.05±0.67^{a}	25.46±0.49^{a}	26.09±2.67^{a}	0.83	0.83	0.94	0.87
Ile	Bitter(-)	90	23.07±4.33^{b}	27.21±6.95^{b}	29.07±7.75^{b}	67.80±1.29^{a}	0.26	0.39	0.43	0.70
Leu	Bitter(-)	190	71.22±5.93^{a}	57.55±3.38^{b}	44.34±4.95^{c}	52.67±1.87^{b}	0.37	0.37	0.33	0.28
Tyr	Bitter(-)	91	67.14±2.67^{c}	86.17±1.49^{b}	91.14±2.38^{b}	108.88±7.72^{a}	0.68	0.87	1.06	1.20
Phe	Bitter(-)	90	73.47±6.30^{c}	106.32±0.77^{b}	111.50±7.28^{b}	137.19±7.77^{a}	0.88	1.16	1.31	1.52
Lys	Bitter/Sweet(-)	50	90.50±2.20^{ab}	75.94±1.14^{c}	88.27±2.77^{b}	94.97±5.11^{a}	1.65	1.40	1.77	1.90
His	Bitter(-)	20	1030.13±21.31^{b}	984.22±52.24^{b}	1005.69±9.57^{b}	1148.75±41.1^{a}	46.81	43.92	50.28	55.07
Arg	Bitter(-)	50	18.83±0.92^{ab}	17.98±0.10^{b}	20.54±1.33^{a}	20.68±1.55^{a}	0.34	0.32	0.41	0.41
Pro	Sweet(+)	50	21.73±1.83^{b}	22.60±2.26^{b}	46.72±1.54^{a}	33.23±13.55^{b}	0.66	1.05	1.23	0.66

Note: TT: taste threshold. Different lowercase letters within the same row indicate significant differences ($p < 0.05$).

Table S5. PLS-DA loadings of FAAs in DMF.

Amino Acid	Loadings 1	Loadings 2	Amino Acid	Loadings 1	Loadings 2
Asp	0.2150	0.0786	Ile	0.4323	-0.0563
Thr	-0.0380	-0.0267	Leu	-0.1919	-0.1574
Ser	0.0027	-0.0155	Tyr	0.3325	0.0582
Glu	-0.0048	0.0030	Phe	0.5256	0.1069
Gly	0.0464	-0.0044	Lys	0.0153	-0.0663
Ala	-0.0707	0.0170	His	0.7079	-0.9641
Cys	0.3406	-0.0330	Arg	0.0041	-0.0024
Val	-0.1855	-0.0890	Pro	0.0795	0.1338
Met	-0.0252	-0.0171			

Table S6. VIP values of VOCs in DMF.

Compound	VIP scores	Compound	VIP scores
Hexanal	3.4064	3-Methyl-2-butenal	0.1205
Ethyl propanoate	3.1688	2-Heptanol	0.0918
2-Methyl-3-(methylthio)furan	2.4237	Pyrazine	0.0898
Nonanal	1.3530	(*Z*)-2-Penten-1-ol	0.0748
Ethanol	1.1704	1-Pentanol	0.0628
Ethyl acetate	1.1133	Benzaldehyde	0.0590
2,5-Dimethylpyrazine	1.0796	2-Methyl-1-propanol	0.0563
Furan	1.0050	(*E*)-2-Pentenal	0.0455
2-Methylpyrazine	0.7515	p-Cymene	0.0335
Pentanal	0.6859	3-Hydroxy-2-butanone	0.0276
Propanol	0.5431	Methyl acetate	0.0175
1-Penten-3-ol	0.3682	(*E*)-2-Hexenal	0.0170
Ethanal	0.3651	3-Methyl-2-butanol	0.0141

2-Methyl-2-cyclopenten-1-one	0.2736	2-Methylbutanal	0.0139
1-Penten-3-one	0.2002	2-Butanone	0.0133
Butanal	0.1921	β-Myrcene	0.0107
2-Propanone	0.1818	(*E*)-2-Heptenal	0.0096
2-Methylpropanal	0.1709	Methyl-2-methylbutanoate	0.0007

Table S7. Correlation coefficients between FAAs and VOCs in DMF.

	Hexanal	Ethyl propanoate	2-Methyl-3-(methylthio)furan	Nonanal	Ethanol	Ethyl acetate	2,5-Dimethylpyrazine	Furan
Asp	-0.26	-0.42	0.72**	0.57	-0.20	-0.95**	0.61*	-0.787**
Thr	0.46	-0.74**	-0.25	0.33	0.38	-0.13	-0.01	0.23
Ser	0.25	-0.76**	0.09	0.23	0.00	-0.46	0.52	-0.14
Glu	-0.02	-0.56	0.33	0.59*	0.24	-0.60*	0.20	-0.39
Gly	-0.30	-0.28	0.69*	0.15	-0.40	-0.79**	0.88**	-0.71*
Ala	-0.76**	0.51	0.55	0.00	0.31	-0.19	-0.10	-0.37
Cys	-0.10	-0.38	0.63*	0.26	-0.66*	-0.85**	0.85**	-0.67*
Val	0.53	-0.01	-0.87**	-0.43	0.37	0.91**	-0.53	0.92**
Met	0.02	0.24	-0.16	-0.40	-0.24	0.38	0.25	0.18
Ile	-0.04	-0.34	0.55	0.11	-0.64*	-0.76**	0.82**	-0.59*
Leu	0.45	0.12	-0.70*	-0.54	-0.02	0.76**	-0.25	0.67*
Tyr	-0.38	-0.18	0.85**	0.35	-0.59*	-0.95**	0.82**	-0.89**
Phe	-0.46	-0.10	0.89**	0.37	-0.55	-0.93**	0.77**	-0.91**
Lys	0.53	-0.87**	-0.13	0.30	-0.05	-0.33	0.46	0.00
His	0.07	-0.45	0.32	-0.10	-0.44	-0.51	0.81**	-0.26
Arg	0.04	-0.55	0.24	0.25	0.18	-0.55	0.34	-0.25
Pro	-0.09	-0.47	0.35	0.64*	0.19	-0.59*	0.29	-0.51
Furan	0.49	-0.05	-0.86**	-0.42	0.54	0.89**	-0.66*	1.00
2,5-Dimethylpyrazine	-0.26	-0.17	0.63*	-0.09	-0.67*	-0.66*	1.00	-
Ethyl acetate	0.28	0.33	-0.77**	-0.55	0.43	1.00	-	-
Ethanol	0.06	-0.12	-0.42	0.07	1.00	-	-	-
Nonanal	0.26	-0.56	0.12	1.00	-	-	-	-
2-Methyl-3-(methylthio)furan	-0.80**	0.29	1.00	-	-	-	-	-
Ethyl Propanoate	-0.70*	1.00	-	-	-	-	-	-
Hexanal	1.00	-	-	-	-	-	-	-

Note: ** indicates highly significant correlation ($p < 0.01$), * indicates significant correlation ($p < 0.05$).
